# Canadian Resources for Siblings of Youth With Chronic Health Conditions to Inform and Support With Healthcare Management: A Qualitative Document Analysis

**DOI:** 10.3389/fresc.2021.724589

**Published:** 2021-10-05

**Authors:** Linda Nguyen, Hanae Davis, Samantha Bellefeuille, Jessica Havens, Susan M. Jack, Briano Di Rezze, Marjolijn Ketelaar, Jan Willem Gorter

**Affiliations:** ^1^School of Rehabilitation Science, McMaster University, Hamilton, ON, Canada; ^2^CanChild Centre for Childhood Disability Research, McMaster University, Hamilton, ON, Canada; ^3^Sibling Youth Advisory Council, Hamilton, ON, Canada; ^4^School of Nursing, McMaster University, Hamilton, ON, Canada; ^5^Department of Health Research Methods, Evidence, and Impact, McMaster University, Hamilton, ON, Canada; ^6^Offord Centre for Child Studies, Hamilton, ON, Canada; ^7^Centre of Excellence for Rehabilitation Medicine, University Medical Center Utrecht and De Hoogstraat Rehabilitation, Utrecht, Netherlands; ^8^Department of Pediatrics, McMaster University and McMaster Children's Hospital, Hamilton, ON, Canada

**Keywords:** chronic health condition, siblings, qualitative analysis, pediatrics, family-centered services, healthcare management, transition to adulthood

## Abstract

**Background:** As children and adolescents with a chronic health condition (CHC) age and transition to adulthood, many will increasingly assume responsibilities for the management of their healthcare. For individuals with CHCs, family members including siblings often provide significant and varied supports. There are a range of resources in Canada to support siblings of individuals with a CHC, but these resources are not synthesized and the extent to which they relate to healthcare management remains unclear.

**Purpose:** The purpose of this document review was to identify, describe, and synthesize the types of resources currently available to provide general information and healthcare management information about how siblings can provide support to individuals with CHCs in Canada.

**Methods:** Print and electronic resources were systematically identified and retrieved from the websites of organizations, treatment centers, and children's hospitals that are part of Children's Healthcare Canada. Each unique resource was treated as a text document. Documents that met the following inclusion criteria were included: addressed the topic of siblings of individuals with a CHC and written in English. Data were extracted from included documents and qualitative conventional content analysis was conducted. Throughout the process of this review, we partnered with a Sibling Youth Advisory Council.

**Results:** The systematic search yielded 1,628 non-duplicate documents, of which 163 documents met the inclusion criteria. Of the total of 163 documents, they were delivered in the following formats: 17 (10%) general informational products (e.g., booklets, videos) about a CHC and sibling relationships, 39 about support programs and workshops (24%), 34 news articles (21%) that described the roles of siblings, and 6 (3%) healthcare management informational products (e.g., toolkit, tipsheets), 31 blogs (19%) and 39 interviews (24%) with parents and siblings. In the blogs and interviews, siblings and parents described how siblings developed knowledge and skills for healthcare management, as well as their role and identity over time.

**Significance:** This study identified that there are limited resources available about healthcare management for siblings of CHC in Canada. Resources are needed to facilitate conversations in the family about the role of siblings with healthcare management of their sibling with a CHC.

## Introduction

In North America, ~15 to 18% of all youths have a chronic health condition (CHC) (1, 2). The term “chronic health condition” encompasses congenital and acquired diseases, as well as physical and mental health conditions (3). There has been a shift toward providing care for a family of a child with a CHC using a non-diagnostic approach (4). Instead of focusing on a specific diagnosis or condition alone, increasing care, and supports focus on providing comprehensive care to address the holistic needs of the individual and family (4, 5).

Families often express significant concerns about how they can best support their child during the transition from the pediatric to adult healthcare systems (6). During this time, youth with CHCs will need to learn how to manage their healthcare, for example learning how to navigate the process of filling prescriptions, scheduling healthcare appointments, and answering questions from healthcare providers. Typically, support is provided by family members through this transition period. In addition to healthcare management, individuals with CHCs are also exploring their interests and goals, including school, work, and leisure (6–9) and learning to navigate new environments, including healthcare for adults, education, transportation, recreation, and social services (7). Throughout the lifespan, families typically can provide support, given their past experiences coordinating their family member's or child's care and knowledge of their child's strengths, areas of improvement, and goals (10).

In addition to parents, siblings are a part of the family who can provide support for their sibling with a CHC. Within the typical lifelong bonds between siblings, these relationships are highly dynamic and can change over time depending on the needs, roles, and commitments of the whole family (11–13). Each sibling relationship is different with varying levels of emotional closeness, social connectedness, and expectations of each other (14). During childhood and adolescence, sibling relationships are unique as they often live and grow up in their shared home environment where they can act as peers, confidants, or role models (15). At a young age, siblings of individuals with a CHC often recognize that they need to support their family in different contexts (16, 17). When there is future planning involved from the whole family, siblings often feel closer to each other and with their family, and they have a clearer understanding about their role for the future during adulthood (18, 19).

In some families, there may not be discussions about the role of siblings but siblings may be expected to become carers to their sibling with a CHC (20). In 2018, the Siblings Needs Assessment Survey was conducted in Canada among young adults who were ages 20 years or older, who had a sibling with a disability and received a total of 360 responses (21, 22). Siblings described concerns for their sibling's future such as finding employment or living independently (12, 23). Siblings might have worries for new responsibilities, such as guardianship or financial responsibilities, when their parents can no longer be the primary caregivers (13, 24, 25). These concerns can affect the extent to which siblings are involved in the healthcare of their sibling with a CHC.

Typically developing siblings might want to support their sibling with a disability, but they require knowledge and skills on how to do this. There are currently “Sibshops” that are offered across ten countries, including the United States and Canada (26). These Sibshops provide an opportunity for siblings to connect with people with similar experiences and share stories. A survey was conducted to evaluate Sibshops, in which 66% of respondents identified that they learned coping strategies, 75% reported that Sibshops had a positive impact on their adult lives, and 94% stated that they would recommend Sibshops to others (27). Often, one of the goals of Sibshops is to provide a space for siblings to meet and share experiences with other siblings of individuals with a CHC in a recreational setting (28). Some SibTeen sessions are also held for adolescents ages 13–17 years old to offer a community of support (29). Although there are support groups for siblings of individuals with a CHC, such as Sibshops and SibTeen sessions, there are no tailored resources or programs for typically developing siblings to share their concerns about supporting their sibling with a CHC specifically for healthcare management. There are many ways that siblings can provide support to their sibling with a CHC. These can be categorized as: concrete support such as taking on responsibilities and providing assistance; emotional support such as listening and empathizing; advice support such as offering information; and esteem support, such as expressing encouragement (30). Siblings can offer these different types of supports to help their sibling with a CHC manage their healthcare.

Informational needs have been identified to be a critical need for siblings (31). Siblings who wish to have a caregiving role often seek knowledge in how to provide care to their sibling with a CHC, how to navigate disability services, and how to seek supports for themselves (31). While there is information available and advertisements of services for siblings on websites of children's hospitals and organizations, many families and siblings identified that they were not aware of this information (32, 33). Siblings identified that they want to have an open, constructive dialogue with their parents about the future, including expectations and responsibilities (13, 24). Often, siblings had to learn how to care for their sibling on their own as information was not always passed down from parents to the siblings (11, 24).

Informational needs are also increasingly being addressed by individuals, including siblings and their families, through the use of the Internet. Siblings can share their experiences and needs online in various formats, such as blogs. Among the few studies that have analyzed the content of blogs, researchers identified how individuals who write these blogs can share experiences that might be different from what might be shared in a research study. Young adults and families have previously written blogs to document their experiences in healthcare, including their emotions and challenges (34–36). Similarly, blogs written by siblings and families can provide insights into the needs of siblings in order to prepare for their roles with healthcare management. There is a gap with little information known about the types of needs about healthcare management that siblings of individuals with CHC are sharing online.

Individuals may also choose to find information online for various reasons, including medical information, such as options for therapy, treatments and health services (37, 38). Siblings of individuals with a CHC require information on how they can provide support with healthcare management. In the Canadian Sibling Needs Assessment Survey, the majority of respondents across all age groups identified online websites as their preferred method for resources, information and tools (22). Programs, such as Sibshops and SibTeen sessions in North America, are often promoted online and share information about eligibility criteria and registration. In other countries, initiatives to support the needs of siblings of individuals with a CHC include Siblings Australia developed in 1999 (39), Sibs in the United Kingdom in 2001 (40), and the Sibling Leadership Network in the United States in 2007 (41). These initiatives in Australia, the United Kingdom, and the United States have been established for many years, and includes an array of support programs and resources for siblings of individuals with a CHC. In Canada, the Sibling Collaborative was established in 2017 and offers online support groups with some resources such as information about the COVID-19 vaccine, finances, and stories from siblings (42). Despite the availability of many resources, as a team, we have heard from siblings that resources about healthcare are not easily accessible or retrievable in Canada. The resources are often posted on certain websites by children's hospitals and organizations, but the websites are not easy to navigate. In Canada, there is no national systems approach to store resources for siblings of individuals with a CHC. Considering the important and multi-faceted roles that siblings can have, it is important to identify and summarize the different types of resources that are available to siblings of individuals with a CHC.

This review aims to identify and describe:

i. the types of resources currently available in Canada to provide both general information and specific healthcare management information about how siblings can provide support to their sibling with a CHC; and
ii. key topics discussed in resources created by siblings and families.

## Methods

### Integrated Knowledge Translation

An integrated knowledge translation approach was used throughout the process of this review to partner with the Sibling Youth Advisory Council (SibYAC) comprised of six young adults who have a sibling with a disability. The SibYAC were first involved with the idea and concept, as well as the research question of this review. The SibYAC shared their experiences with searching for information to support their roles as siblings, and they identified a need to identify and synthesize resources that are available to siblings of individuals with a CHC. These experiences from the SibYAC provided a clear rationale to support our review aims. There were individual check-in meetings with each SibYAC member, and an engagement framework (43) and Involvement Matrix (44) were used as tools to ask about the tasks and roles that they would like to have in this review. The SibYAC were further involved in data analysis by sharing their perspectives for the retrieved documents to ensure that the extracted data are synthesized meaningfully for siblings, families, and other stakeholders. They were then involved with the interpretation of results and drawing conclusions. Meetings were held with the SibYAC to ask about their reflections of the summary of results with guiding questions including: How do the documents and websites support siblings in their role? Based on the documents and websites, what are some needs, information, or questions that you still have as a sibling? For example, in healthcare or in general. Reflections from the SibYAC helped to identify the gaps and future directions about resources to support siblings in their roles, including with healthcare management of their sibling with a CHC.

### Qualitative Document Analysis

Qualitative document analysis involves a systematic search of documents and resources, which includes both printed and electronic resources (45). A variety of documents can be analyzed, including books, brochures, diaries, journals, event programs, or news articles.

### Search Strategy

A comprehensive search was conducted on publicly available websites of thirty-one organizations, including children's hospitals, and rehabilitation centers that are part of Children's Healthcare Canada (46). These were selected to provide an initial understanding about the types of resources that are available for siblings of individuals with a CHC in a healthcare setting. The websites were searched in August 2020. A broad search strategy was employed in the search engine of each website with the terms: “sibling,” “brother,” or “sister.” All documents from the search were digitally retrieved using a feature called NVivo Capture and imported into NVivo (Version 11.4.3). Duplicates of documents across websites were removed.

### Inclusion and Exclusion Criteria

Text from all retrieved documents was initially scanned in NVivo for the key terms of “sibling,” “brother,” or “sister.” Documents that included at least one of these key terms were read by the first author (LN). Identified documents were included in the review if they: (1) addressed the topic of relationships between siblings with and without a CHC; and (2) were published in English. Documents were excluded if the sibling was mentioned but did not discuss supports of siblings or the relationship between siblings of individuals with a CHC.

### Ethical Considerations

Ethics approval was not required to retrieve and analyze documents that are publicly available on the Internet. An assessment of online documents can be conducted to identify the intent of online documents and its use in research, and documents that are written for public intent do not require consent from the creators or authors of the documents (47). In the analysis of retrieved documents, there was careful consideration to protect the privacy of the creators for the documents, and all personal identifiers were removed from included documents.

### Data Extraction and Analysis

A data extraction template was created using Microsoft Excel Version 16.41 to collect data from each document (48). This template included the following categories: document source, document type, purpose/goals, and key content. For document types coded as “blog” or “interview,” content data for two additional categories were extracted: (1) family characteristics; and (2) CHC of an individual in the family. Additionally, all blogs and interviews were read and re-read in an iterative process to achieve immersion in the data and understand the stories shared by siblings and parents. Conventional content analysis was conducted by the first author (LN) for documents that were coded as blogs or interviews (49). Initial codes were developed based on the full text of the blogs and interviews, and these codes were then organized into categories to depict how they were related and linked to each other. Codes were grouped into meaningful clusters or categories based on their similarities in concepts. An Excel spreadsheet was created, that included extracted quotes and codes that were grouped into categories. Each category was expanded into a short statement to describe the key topic shared by siblings and families. Two analysis meetings were then held with individuals familiar with the content (e.g., SibYAC) and qualitative analysis (e.g., graduate students, co-author SJ) to review and name the categories, and identify additional properties and dimensions of each meaningful cluster. Analytic notes were written by the first author (LN) about how the categories related to each other to form meaningful clusters. While the content of all documents was analyzed to identify information and supports for healthcare management of an individual with a CHC, conventional content analysis allows for the identification of key topics from included documents that describe the experiences of siblings of individuals with a CHC beyond healthcare management. In this review, recognizing that gender is non-binary, we refer to siblings as a “brother” or “sister” based on the information provided in the resources included in this review with the recognition that siblings may identify themselves along a spectrum.

### Data Credibility

To ensure credibility of the data, an audit trail and multiple analyst triangulation were used as two strategies. An audit trial was created to describe the steps and document decisions that were made about data extraction, as well as the identification of codes, categories, meaningful clusters, and key topics identified in the documents (50, 51). Sufficient time was also spent reviewing each source of information to identify recurrent patterns and key topics of the documents (52). The first author (LN) spent extensive time to read and re-read all documents, and took field notes of emerging ideas for each document in an Excel document (e.g., What is the main message about this document? How does this document relate to other documents?). To further enhance the credibility and dependability of the data, the lead author engaged in reflexivity and documented their own biases, preferences, and preconceptions about the topic in a series of memos (53, 54). Analyst triangulation was employed, in which multiple individuals with different backgrounds and expertise offered their perspectives about the preliminary and final findings (54). Two initial meetings were held to review and discuss how to organize preliminary findings: first with a group of graduate students with expertise in mixed methods and qualitative research, and then with the SibYAC. Two additional meetings were held with the SibYAC to share their reflections about the meaning of the findings in this review to them, describe whether the key topics from the blogs and interviews resonated with, or differed from, their experiences as young adult siblings of individuals with a disability, and identify gaps for future directions. All SibYAC members present at the meeting described that the key topics were similar to their experiences, and they provided suggestions for future directions in the development and enhancement of resources for siblings of individuals with a CHC. All authors of this review are from a multidisciplinary backgrounds including cognitive psychology, education, nursing, occupational therapy, physiatry, rehabilitation, patient-oriented research, and lived experiences, and all provided their perspectives on the synthesis of findings.

## Results

**Figure 1:**
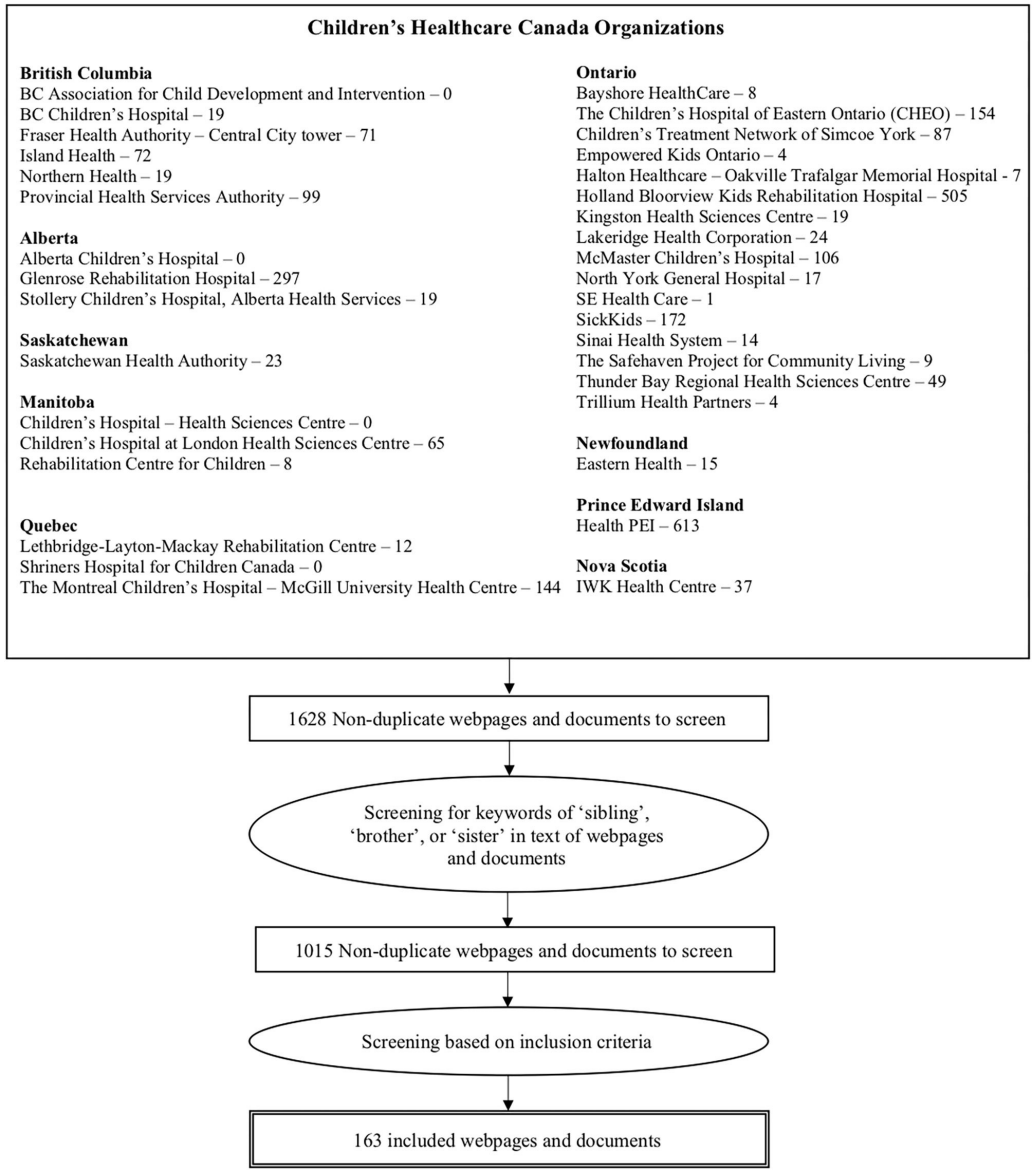
Flow diagram outlining the section of included websites and documents.

The systematic search yielded 1,628 non-duplicate documents and resources, with 1,015 documents and resources that included keywords of “sibling,” “brother,” or “sister.” There were 163 documents and resources that met the inclusion criteria (See Figure 1).

All resources were identified from treatment centers that provide inpatient and outpatient services to children and adolescents with a CHC. Some documents discussed CHCs as a broad group of conditions, while others referred to specific conditions such as autism spectrum disorder, cerebral palsy, Down syndrome, epilepsy, genetic disorders, juvenile arthritis, intellectual disorders, or mental health disorders. The documents included 6 books (55–60), 1 podcast (61), 34 programs and 5 workshops [references provided to websites where ongoing programs (62–67) and workshops (63) are advertised], 7 films and videos (68–74), and 34 news articles. Among the 34 news articles, they referred to 2 announcements (75, 76), 6 “awareness” recognition of different days or months of CHCs (77–82), 5 events (82–86), 10 research studies (87–94), 4 participation in research [references provided to websites where active research studies are posted (95, 96)] and 7 stories (97–103). Three documents referenced a toolkit (104–106), 3 booklets (107–109), and 3 tip sheets (110–112). Of the total of 163 documents, there were 31 blogs (19%), of which 13 (8%) written by parents and 18 (11%) written by siblings, and thirty-eight interviews (23%) with 12 (7%) interviews with parents and 26 (16%) interviews with siblings, and one interview with a family that included both the parents and siblings. Table 1 provides details about the document type, source of documents, number of documents and references, target audience, purpose and goals, and summary of key content.

**Table 1:** Description of documents.

**Document type**	**Source of documents**	**Number of documents and references**	**Target audience for resource**	**Purpose and goals**	**Summary of key content**
**Resources about general information**					
Booklets	One hospital in Ontario and one service provider in Prince Edward Island	3 (107–109)	Parents and educators of an individual with a CHC	To offer strategies for parents and educators of an individual with a CHC and their siblings	Two booklets, one for parents and one for educators of individuals with autism spectrum disorder, included a booklist relevant for siblings to learn about autism. One booklet offered strategies for educators to support siblings of individuals with childhood cancer
Books	Two hospitals in Ontario	6 (55–60)	Public	To share about the importance of relationships of siblings, when a sibling has a CHC	The books recognize how there is a needed space and role of siblings
Podcast	One hospital in Ontario	1 (61)	Public	To tell the stories of people with a CHC, including the experiences of siblings in families with a child with a CHC	One podcast spoke about the stories of siblings of people with a CHC
Programs	Four hospitals and two service providers in Ontario	34^*^ (62–67)	Parents/caregivers and siblings of an individual with a CHC	The purposes of the programs included providing opportunities for families, including siblings, to connect with other families of individuals with a CHC, spend together as a family through various community events, providing support to siblings to understand the CHC and develop coping strategies	Programs included: •Family Nights held on a regular basis (e.g., monthly) for siblings and families of individuals with a CHC to connect with each other •Informal family playgroups that have regular events (e.g., monthly) for families to participate in the community, e.g., indoor playground, escape room, arts and crafts, board games •Support program, including the Sibshops with sessions for siblings with a range of ages from 7–25 year olds to support siblings who may have questions or are seeking coping strategies with their brother or sister with a CHC •Bravery Beads Program that allows children an opportunity to collect a different bead for each procedure or event while visiting the hospital for a treatment. An example of how the beads were used between siblings, where the sister educated the class on what her brother went through and still has to go through
Workshops (one-time event)	One hospital in Ontario	5^*^ (13)	Siblings and parents of a child with a CHC	The purposes of the workshops were to provide opportunities for siblings to connect with other siblings, discuss ways that parents can support siblings of individuals with a CHC in the family, and advertise other ongoing programs for siblings and families	An opportunity for parents and siblings (either in separate groups or together) to ask questions, including to a panel of adult siblings about their experiences. Workshops were provided both online and virtually
Films and videos	One hospital in Ontario	7 (68–74)	Public	To share about the experiences of families, including the perspectives of siblings and parents, about growing up with a child with a CHC	The types of CHCs covered in the films and videos included autism spectrum disorder, Down syndrome, brain damage, and Type 1 diabetes
**News articles**					
Announcements	One hospital in Ontario	2 (75, 76)	Public	To share information about results, which included a donation to create a center in Canada to support adults with disabilities, a partnership to build rehabilitation capacity for children with a CHC between institutions, and the winner of a “filmpossible” award	Donation and partnership were inspired by the experience of a family with an individual with a CHC. Stories about the experiences of siblings of an individual with a CHC shared in the announcement
Awareness about CHCs	Two hospitals in Ontario and one hospital in Quebec	6 (77–82)	Public	To raise awareness on specific days to appreciate different disabilities and roles, in which siblings have a part in the awareness of CHCs	Each document described the awareness of different days: •Sibling Appreciation Day •Purple Day for Epilepsy •Cerebral Palsy Awareness Month •Childhood Cancer Awareness Month •Children's Grief Awareness Day •International Day of Persons with DisabilitiesTo raise awareness of these different days, siblings shared stories about their brother or sister with a CHC
Events	One hospital in Ontario and one hospital in Quebec	5 (82–86)	Public	To raise awareness about the stories and roles of siblings	Events included advocacy for individuals with disabilities, as well as events for siblings to attend
Research Studies	Two hospitals in Ontario and one hospital in Quebec	10^**^ (87–94)	Public	To share the findings of research studies	The topics of research studies included genetic information for siblings of individuals with ASD, genetic mutation that lead to lymphoma, survey findings about the experiences of siblings of a brother or sister with a developmental disability, successful donor liver transplant between siblings, rare autoinflammatory disease based on research of two siblings with juvenile idiopathic arthritis, immunotherapy, trials about accessible equipment, and accessible video games for children with disabilities
Participation in research	One hospital in Ontario and one health center in Nova Scotia	4^*^ (95, 96)	Public	To recruit participants for a research study	The topics of the research studies were assessments of early behavioral signs of autism spectrum disorder in infants and the experiences of sibling including their needs and feelings
Stories	Health service provider in British Columbia, three hospitals in Ontario	7 (97–103)	Public	To share the stories of families who have an individual with a CHC	Stories were shared by mothers and siblings about their experiences with a person with a CHC. For some sibling relationships, both siblings had a CHC, and shared how they supported each other
**Resources for how siblings can provide support with healthcare management**					
Toolkit	One hospital in Ontario	3 (104–106)	Siblings of an individual with a CHC	To offer strategies for siblings of individuals with an acquired brain injury	All documents referred to one toolkit, the SibKit 1.0 (105), which provides information about strategies for siblings of individuals with an acquired brain injury
Tip sheets	One hospital in Ontario and one hospital in British Columbia	3 (110–112)	Parents and siblings of an individual with a CHC	To offer strategies for parents to support siblings, and for siblings to support their sibling with a CHC	One tip sheet described how parents can support siblings of an individual with a CHC. Two tip sheets offered strategies for siblings to cope with the surgery of their brother or sister, as well as, when their brother or sister is an inpatient
**Interviews**					
With Siblings	Three hospitals in Ontario and one hospital in Quebec	26^***^	Public	To share about the experiences of siblings of a child with a C	Key topics discussed during the interviews and blogs are presented in Table 2
With Parents	One hospital in Ontario	12^***^	Public	To share about the parents' experiences when they have multiple children including a child with a CHC	
**Blogs**					
Written by siblings	Three hospitals in Ontario	18^***^	Public	To share key messages from siblings about their experiences when they have a sibling with a CHC	
Written by parents	Two hospitals in Ontario and a health service provider in British Columbia	13^***^	Public	To share the stories of families of a child with a CHC and the siblings from the parents' perspective	

* *Programs, workshops, and participation in research studies are advertised and updated on an ongoing basis on the websites of the children's hospital and/or treatment centers. The number of documents refers to the advertisements and newsletters that was posted on the websites*.

** *Two documents are no longer available on the website, but was included in the analysis*.

*** *wReferences were not provided in order to ensure confidentiality of the authors and families mentioned in the blogs and interviews*.

### Types of Resources for General Information

The majority of resources were general informational products for siblings of individuals with a CHC, which are available in a variety of formats. Supplementary Table 1 presents detailed descriptions of these resources.

*i. Booklets and books*. Books were available for siblings and families, in which some highlighted the need to understand the importance of sibling relationships, for example, creating a space for siblings to understand their emotions when they have a sibling with a CHC. Booklets were also available to provide guidance to parents and teachers about how to communicate with siblings of someone with a CHC.
*ii. Podcasts*. Personal stories from families, including siblings, were shared through podcasts, films, and videos. These stories described the journey of the whole family, and one podcast discussed the relationship between the siblings in which one sibling has a CHC.
*iii. Programs and workshops*. There are advertisements that announced past programs (*n* = 34) and workshops (*n* = 5) available to siblings and families. Among these 39 documents, 17 were in-person, 8 were virtual due to COVID-19, and 14 did not indicate the type of format. Most programs offered were sibling support groups, such as Sibshops, that are available throughout the year for siblings who are ages 7–25 years old. There were programs specifically for families to connect with other families of children with autism spectrum disorder available for free [reference to ongoing advertisement about the program (57)].
*iv. News articles*. All stories that were published as a news article were authored either by parents (*n* = 1), mothers (*n* = 2), or a sibling (*n* = 3), both a mother and sibling (*n* = 1). Articles authored by mothers focused on stories of their child's lived experience, parenting multiple children with a CHC or the same CHC, and/or the roles that other children may assume when there is a child with a CHC. Siblings discussed topics, such as sharing their emotions about their sibling relationship, providing support with healthcare management, and transitioning into different roles as a sibling, such as becoming a caregiver. Throughout the year, there were news articles with announcements about initiatives that were inspired by the stories of siblings. For example, there were announcements about various “awareness” days and months about specific disabilities and health conditions, which provided an opportunity for siblings to share stories about their sibling with a CHC (69–74). News articles also advertised research studies that were completed or actively recruiting sibling participants. The topics of these studies included genetic studies for specific health conditions, such as autism spectrum disorder and lymphoma, successful organ transplants between siblings, effectiveness of assistive equipment, and a survey to understand the needs and feelings of siblings of youth with a CHC.

### Type of Resources for How Siblings Can Provide Support With Healthcare Management

There are few resources that provided information for siblings about their roles with respect to the healthcare management of their sibling with a CHC. When resources were available, they were formatted as either tip sheets or as a toolkit.

#### Toolkit and Tip Sheets

Both parents and siblings could refer to different sheets that were available for download online, which included tip sheets (110–112), and a toolkit (104). These sheets also focused on strategies for how siblings can provide support to their sibling with a CHC. For example, there was a tip sheet that described strategies for siblings of inpatients at a children's hospital (112). Some strategies for how siblings can be included as part of the inpatient stay were being a part of their sibling's care team, doing fun activities together, talking to their sibling, and helping the sibling to decorate their room (112). While the resources primarily focused on providing knowledge about a CHC to siblings, some resources provided additional strategies for siblings to support the healthcare management of their sibling with a CHC. A toolkit was also co-designed with siblings, clients, parents and clinical staff for brothers and sisters of children who have an acquired brain injury (104). The toolkit was described as a resource that siblings can use to learn knowledge about their sibling with an acquired brain injury and learn how to explain this injury to other adults who can provide help, when needed (104).

### Blogs and Interviews

**Figure 2:**
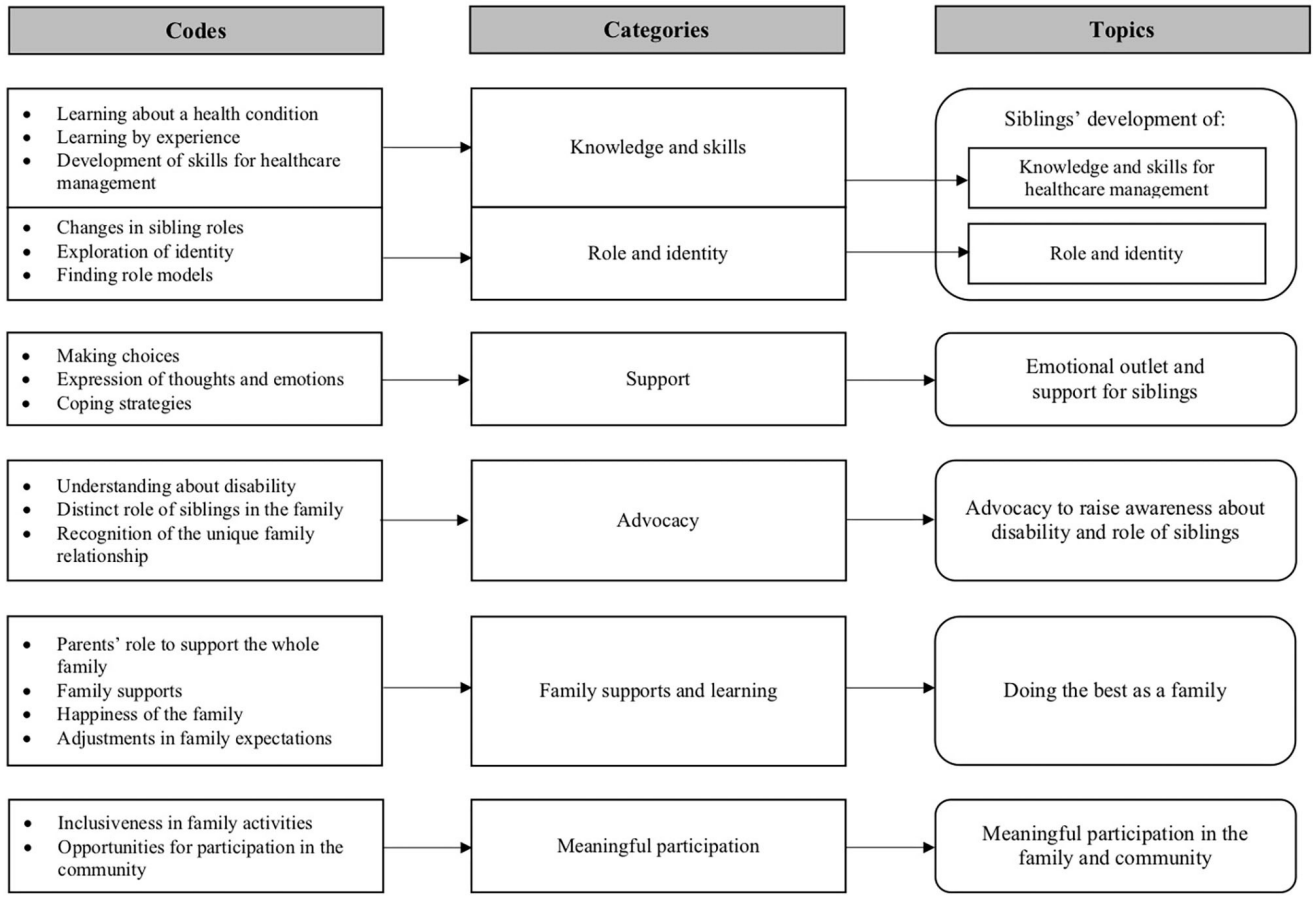
Codes, categories, and topics indentified the blogs and interviews.

Siblings and families described different types of CHCs in blogs and interviews. Some siblings also had the same CHC as other siblings in the family. The age of siblings and individual with a CHC discussed in blogs and interviews ranged from infancy to older adults. The size of families ranged from one to five children. Based on an analysis of the content from blogs and interviews shared by siblings and parents, a conceptual map was developed to describe the codes, categories, and key topics discussed in these documents (See Figure 2). Detailed descriptions about these key topics are described in detail below. The frequency that these topics were identified in the blogs and interviews are provided in Table 2.

**Table 2:** Frequency of topics identified in interviews and blogs with parents and siblings.

**Topics**		**Siblings**		**Parents**	
		**Blogs**	**Interviews**	**Blogs**	**Interviews**
		**(*n* = 18)**	**(*n* = 26)**	**(*n* = 13)**	**(*n* = 12)**
Siblings' development of:	a) knowledge and skills for healthcare management	7	8	4	5
	b) role and identity	12	16	4	6
Emotional outlet and support for siblings		7	12	–	3
Advocacy to raise awareness about disability and role of siblings		6	3	–	–
Doing the best as a family		–	2	5	6
Meaningful participation in the family and community		6	4	–	5

*–Topic was not identified*.

* *Multiple topics may be identified in each interview and blog*.

#### Siblings' Development of Knowledge and Skills for Healthcare Management

Siblings described how they needed to learn about the meaning of disability. Some siblings did not understand specific CHCs, such as the different treatments and services their siblings had to receive to manage their CHC. Siblings often described that they simply saw their sibling for who they were, regardless of the CHC. Parents shared stories in their blogs about the forming of relationships between their children. Young children learned how to develop their relationship with their sibling with a CHC. A mother shared the story of how she saw her two children interact with each other, where her young daughter asked to hug her brother or hold his hand when he was using an assistive device to walk. As siblings began to develop an understanding about the CHC, some siblings offered support with healthcare management. For example, a mother described how her daughter learned to be present and hold her brother's hand when he was using a suction machine. Siblings shared in interviews about how they learned different ways to support their siblings. For example, a sister observed her mother apply breathing techniques with her brother and she learned how to do the same. There was a process in which siblings first needed to learn about the CHC and develop a relationship with their sibling with a CHC, which then allowed them to learn how to offer support with healthcare management.

#### Siblings' Development of Role and Identity

The role of being a sibling to someone with a CHC provided them with experiences about a CHC, and the sibling role became a part of their identity. Young adult siblings shared in written blogs about how they were developing their own identity, such as moving away for university and developing their career. For some siblings, the experience of growing up in a family of an individual with a CHC motivated them to pursue a career to support other children with a CHC, such as healthcare professions and research about a CHC. Siblings would bring their personal experiences about a CHC into their professions, such as an understanding about a CHC in research or how to interact with families. Their personal experiences about a CHC also motivated them to use their academic knowledge to create resources, such as mobile applications or tools that children with a CHC could use. Both parents and siblings identified multiple roles that siblings had in the family. Siblings continued to maintain a close relationship, and when one sibling had an acquired CHC, the siblings would learn how to provide support to each other. For example, a sibling described how he went to therapy appointments with his brother who had an acquired brain injury. The sibling provided both support and humor by being present at the therapy appointments, and the parents described how the sibling became a part of the care team. Adult siblings described challenges that they had when they became a caregiver, such as the sacrifices that they had to make with living with their sibling with a CHC or not being able to work full-time.

#### Emotional Outlet and Support for Siblings

Siblings wrote in blogs that they shared with the community about both the positive aspects of their relationship with their sibling with a CHC as well as the challenges. Siblings shared the message about how they are not alone, where their thoughts and feelings matter. Some siblings pursued their own goals and happiness, and also chose their roles with their sibling with a CHC. Some adult siblings experienced guilt when they did not voice their opinions. One adult sibling shared her sense of guilt when her brother was sent to an institution that the family believed was a good option at the time. Siblings spoke about how their emotions were connected to the emotions that their parents were experiencing, such as frustrations and stresses. Some siblings wanted to find ways to address the challenges such as learning how to help their sibling with a CHC. With the different emotions that siblings were feeling, they sought ways to have an outlet to express their emotions. A Photovoice program was offered to siblings and young patients with cancer, where they took photographs that represented their experiences that were later displayed at an event for the public community. Siblings were developing skills in how to cope with their emotions, and parents identified how these siblings would develop personal skills such as being caring and empathetic. Siblings also shared about the importance of open communication with parents because siblings might have hidden emotions. Siblings identified that they might not initiate discussions with parents about their feelings, and parents should create a space for these discussions.

#### Advocacy to Raise Awareness About Disability and Role of Siblings

Some siblings became advocates, in which they expressed a need to explain what disability was to their peers. For example, a sibling of a brother with autism described how she read a book to her class to explain autism and she often took the time to answer questions from her classmates. Siblings valued the connection that they had with other siblings who had similar experiences. Some siblings grew up without knowing about other siblings who have a sibling with a CHC. Siblings wanted to connect to a community of siblings to not only advocate for CHC and disability awareness and supports, but also learn about the role that siblings can have. For example, some siblings did not realize that they developed skills that could be well-suited for healthcare professions. One sibling described how she learned about the profession of a child health specialist after connecting with another sibling. Furthermore, adult siblings who were caregivers or guardians of their sibling with a CHC they identified how their roles were often not recognized at work. For example, employers recognized when co-workers needed to leave to take care of their child but not for their sibling with a CHC. Siblings identified how there should be recognition of the important role that they have. They all wanted to be part of a community where they can create change and advocate for a diverse community that their own sibling with a CHC could meaningfully participate in.

#### Meaningful Participation in the Family and Community

Families identified the importance of creating an inclusive environment where a child with a CHC can participate in activities. Some families planned trips and made sure that they rented adaptive equipment to ensure that their child with a CHC could participate in activities, such as hiking, biking, or kayaking. In their daily lives, young siblings shared in their blogs that they made sure that their sibling with a CHC was included in the games that they played with their friends. One family thought about different ways that every member of the family could participate in activities. When a sibling might be attending speech therapy, other family members could coordinate to have the other siblings participate in a sports activity at the same time. Parents identified how it was important to make sure that all siblings could meaningfully participate in the community. Both parents and adult siblings expressed their concerns about opportunities for their sibling with a CHC to participate in the community in the future. Siblings shared the positive value of a job for their sibling with a CHC, which provided a sense of pride to participate in the community. Some siblings wanted to address concerns about how to create an inclusive community for people with CHCs, and they created mobile applications to encourage their sibling with a CHC to develop the skills needed to participate in the community. For example, one sibling created a mobile application with a set of cards with which an individual with autism could practice the skills they needed to carry out an activity, such as taking public transportation. Both parents and siblings sought opportunities for a sibling with a CHC to participate in the community as they grow older.

#### Doing the Best as a Family

During separate interviews, parents shared about how they were doing the best that they could as a family and siblings shared how the journey of every family was different. A mother shared in her blog about experiences with raising her children, including children with a learning disability and Down syndrome, and she needed to time to learn about her children. For other parents, they learned about the different types of supports that would be appropriate for their child with a CHC and there was no “one size fits all” approach. Some parents initially chose to keep their life private, and they did not want to burden others with the responsibilities in caring for their child with a CHC that they feel were their own. They gradually recognized how it was important to reach out to others for support, such as their children and neighbors. Some parents also sought respite services to take care of their own health in order to optimize the care that they could provide to all of their children. In addition to services, parents described the value of building a network of supports, such as connecting with other families with similar experiences. They wanted to have opportunities to meet other families and participate in activities that included the whole family. Some families created videos and films to share their story of both the positive experiences and challenges with other families.

### SibYAC Reflections on the Findings

After synthesizing the findings from this review, the SibYAC members were asked to share their perspectives about the meaning of these findings. There were key topics raised in the blogs and interviews included in this review, and the SibYAC members were asked about whether these topics resonated with their own experiences of siblings of individuals with a disability. Siblings who wrote the blogs and inteviews included in this review identified that they wrote blogs as a way to share their stories so that other siblings would know that they are not alone, and writing blogs was an outlet for their emotions. Similarly, SibYAC members also wrote personal blogs about their personal experiences as a sibling and the roles that they have had. One SibYAC member shared an excerpt of her journal while her brother, who has cerebral palsy, was in a rehabilitation hospital after orthopedic surgery: “As my brother began to see progress into the next day, so did I. As he found a rhythm and learned the shuffles of the hallway, so did I. And before I knew it, I fell head over heels into the routine of physical and psychological exhaustion but unimaginable emotional fulfillment.” She shares that her personal experience is a clear example of why consciously integrating siblings into the family-centered care model is so important.

While the findings of this review help to identify key resources for general information and information of how siblings can provide support with healthcare management, the SibYAC continued to identify that there is a need for advocacy to raise awareness about the important roles that siblings have. They often had to learn to develop knowledge and skills, in order to have a role with supporting their sibling with a disability with healthcare management. A SibYAC member shared: “There is no handbook for special needs siblings. It's not something that's majorly talked about and kind of always felt like a big secret. Every day, I am learning more about how to appropriately support my sibling through the transition from pediatric into adult healthcare.” While this review identified that there are resources available for siblings of individuals with a CHC, few resources offer support for how siblings can be involved with the healthcare management of their sibling with a CHC.

As the SibYAC reflected on these findings, there is a critical gap in which there are no online resources available from Children's Healthcare Canada to support siblings in conversations about healthcare management and future planning to their sibling with a CHC, even though many siblings might already be part of the care team. A SibYAC member shared her personal experiences with this gap: “There has never been planning about the present, day-to-day things, let alone future planning, that has included me. The extent that I have been involved with my siblings is equal to the amount of intention and force I used to create a space for myself.” The SibYAC shared how discussions about future planning can be helpful for families to ensure that there is clear communication about the role of siblings.

## Discussion

This review identified a variety of resources and documents available in English for siblings and families of children with CHCs across organizations of Children's Healthcare Canada. Most resources consisted of general information for siblings and families: to become aware and learn about different CHCs through books, news articles, and podcasts. There is an increasing trend in the use of the Internet for health information among patients, their families and general public (113), and each family requires different types of information based on their needs (114). In a qualitative study to explore the experiences of parents of children with disabilities who sought information, parents used online information to supplement the information provided by professionals (38). When healthcare professionals did not provide enough information during a consultation, some parents searched on websites of hospitals and rehabilitation centers for additional medical information (38). In the search for information, parents may also identify resources for how they can support the siblings of a child with a CHC. This review retrieved booklets for parents about how to communicate with siblings of children of individuals with a CHC.

In this review, few resources were identified to support siblings in the role of healthcare management with their sibling with a CHC. Similar to parents, siblings might also have questions and would like to have more information to support their roles in the healthcare management of their sibling with a CHC. Siblings of individuals with a CHC require skills and knowledge if they choose to have an active role in the healthcare management their sibling with a CHC. There are tip sheets that provided guidance for siblings to build a relationship with their sibling with a CHC, such as playing games or doing activities, as well as how to be a part of the care team (111, 112). There is also a toolkit that originally developed for siblings of individuals with an acquired brain injury, recently expanded to include different disabilities (105). It is important to consider and tailor different resources to prepare for the roles that siblings might choose to have, including with healthcare management of their sibling with a CHC.

This review highlighted a key gap in the needs of siblings based on the personal stories that they shared through blogs; many identified a need for emotional support. Blogs can be helpful for siblings, where they might find comfort to know that they are not alone (115). Siblings require acknowledgment of their emotions, and for some siblings, they are learning how to address their emotions as they continue to have a role in healthcare to their sibling with a CHC. Siblings might choose to seek online support to be part of a community with others who have similar experiences (115). Parents and families have previously described the importance of being a part of an online community where they can seek resources and connect with other families (38, 116), and siblings might have similar motivations to connect with other siblings online.

Siblings also shared, in blogs and interviews, their perspectives about the importance of advocacy to raise awareness about CHCs and their own roles. Siblings may need to advocate for their role in the family. This review identified that siblings require additional resources in order to learn and be prepared for their future roles. The extent of discussions about future planning can vary in families, and siblings are often not included in these discussions (13, 117). While some parents may wish for the siblings to have their own separate lives, siblings shared in qualitative studies that they chose to have active roles such as being a caregiver and they identified the need to have conversations with their parents about future planning (118, 119). Discussions about future planning can be helpful for families, providing an opportunity for siblings to identify new or changing roles and to facilitate the sharing of information between parents and siblings (31, 33). These discussions can be ongoing to adapt to the changing situations of the family over time. While these discussions can be challenging and complex for families, siblings identified the need to have clear plans so that they can be prepared for their future roles (117, 119, 120).

Many families shared how they are learning from experience and doing the best that they can with their family member with a CHC. In this review, both parents and siblings described the positive value of creating a supportive and inclusive environment of a person with a CHC. This inclusive environment applied to the family environment where some families sought opportunities to participate in different activities, as well as in the community such as having a job. Both parents and siblings described the concern that they had for an individual with a CHC after graduating from high school, and they were worried that there may be fewer opportunities to participate in the community. This concern about the transition to adulthood for individuals with a CHC has been raised in the literature (121, 122), and current news noting that there are ~12,000 young and middle-age adults with disabilities in Ontario, Canada who are on a waiting list to seek supports and residential care (123). In addition to employment opportunities, both parents and siblings have future worries for their child with a CHC such as the navigation from pediatric to adult healthcare services (122, 124). Some siblings shared in blogs and interviews how they gradually learned to take on caregiving responsibilities. Siblings are becoming adults and they may take on future caregiving responsibilities. They are often learning through experience about how to care for their sibling with a CHC throughout the lifespan (119).

### Strengths and Limitations

A strength of this review is the involvement of the SibYAC as advisors throughout the process of this review. They provided their perspectives on the aims of the review, data analysis of resources, and future directions on how to disseminate the findings and develop future resources. Another strength of this review is that the resources identified have been compiled and can be applied to enhance existing resources for siblings of individuals with a CHC and inform the co-development of future resources. A limitation of this review is that the information that can be extracted from the documents included may be restricted by the purpose of the document and the content that the creators choose to share. Another limitation is that all documents were identified from children's hospitals and treatment centers that are part of Children's Healthcare Canada were in English and excluded documents in French. The documents and resources from the websites of organizations that are part of Children's Healthcare Canada might be selectively published and might not include information about the care that these organizations provide. The information may not be reflective of the entire landscape of resources for Canadian siblings of individuals with a CHC. In addition, at the time of this review, documents and resources were retrieved from 31 organizations that were a part of Children's Healthcare Canada. Since then, eight additional organizations have been included. Most organizations that are part of Children's Healthcare Canada are children's hospitals and rehabilitation centers, and resources from services offered in the community, such as mental health services, might have been missed. However, the resources and documents in this review provided a starting point for identifying general information and information about how siblings can support their sibling with healthcare management. Additionally, while data extraction and coding was conducted by a single analyst which limits our ability to report on inter-coder reliability, the categories and meaningful clusters of data that were developed were reviewed and discussed by two key stakeholder groups, a form of analyst triangulation and peer debriefing that enhances overall data credibility.

### Future Directions

This review highlighted key gaps that can be addressed in the future in order to optimize supports for siblings of individuals with a CHC. First, access to existing resources for siblings should be improved by compiling and storing them in one place. As knowledge translation and dissemination can include multiple strategies, there can be multiple formats of resources created, such as infographics, toolkits, videos or podcasts. The Health Hub in Transition in Canada (125) and the F-words for Child Development Knowledge Hub (126) are examples of where information and tools are available online. The uptake and impact of the knowledge translation and dissemination strategies should be evaluated. Second, there should be resources to support siblings in the healthcare management of their sibling with a CHC. In this review, both parents and siblings shared in blogs and interviews the important role of siblings in healthcare management. Despite the important role that siblings might want to have with healthcare management, there are few resources available to support and empower siblings in this role. Third, this review identified that there are no resources in English available within the online materials from the organizations through Children's Healthcare Canada for parents or siblings to facilitate ongoing conversations about the roles that siblings would like to have with their sibling with a CHC. The conversations could also include the topic of healthcare transition about how youth, siblings, families, and healthcare professionals can help youth prepare for the transfer to adult healthcare (127). Tools could be developed to facilitate these discussions in the family and with healthcare professionals (32, 118). Finally, resources could be developed for other professionals, including teachers and healthcare providers, to encourage discussions about the experiences and roles of siblings beyond healthcare management. Siblings have identified that they wanted more information about future responsibilities, such as legal and financial information regarding the care of their sibling with a CHC (22, 24).

## Conclusion

This review identified resources for siblings that are available from children's hospitals and organizations that are part of Children's Healthcare Canada. Resources that are available for siblings of individuals with a CHC mainly address general information, such as support programs and workshops. There are some resources, such as tip sheets and a toolkit, to offer strategies for siblings to learn about the healthcare management of their sibling with a CHC but these resources are only available at two children's hospitals. Siblings shared about their experiences in blogs and interviews, including their development of knowledge and skills for healthcare management, as well as roles and identity that often relate to the healthcare management of their sibling with a CHC. There is a key gap in available resources, in which siblings and parents identified that knowledge and skills for healthcare management is an important role for siblings but there are few resources that provide this information. Given the needs expressed by siblings, future resources should be developed to share information about healthcare management for siblings, as well as tools to facilitate family discussions about the roles that siblings would like to have in the future. A synthesis of the identified resources could be shared in an accessible format, such as in an online hub, for siblings and families.

## Author Contributions

LN, BDR, SJ, MK, and JWG contributed to the conceptualization and design of this review, and perspectives from HD, SB, and JH informed the review aims. LN drafted the manuscript. HD, SB, and JH contributed to the draft of the Reflections section. All authors provided input for the analysis, interpreted the findings, revised and reviewed the manuscript, and provided their final approval.

## Funding

This review was supported by the Canadian Institutes of Health Research Patient-Oriented Research Award—Transition to Leadership Stream—Phase 1 held by LN. The partnership with the Sibling Youth Advisory Council was financially supported by the Graduate Student Fellowship in Patient-Oriented Research through the CHILD-BRIGHT Network held by LN. During the work presented in this article, the Scotiabank Chair in Child Health Research was held by JWG.

## Conflict of Interest

The authors declare that the research was conducted in the absence of any commercial or financial relationships that could be construed as a potential conflict of interest.

## Publisher's Note

All claims expressed in this article are solely those of the authors and do not necessarily represent those of their affiliated organizations, or those of the publisher, the editors and the reviewers. Any product that may be evaluated in this article, or claim that may be made by its manufacturer, is not guaranteed or endorsed by the publisher.
