# Supplementary material for: Canadian Resources for Siblings of Youth With Chronic Health Conditions to Inform and Support With Healthcare Management: A Qualitative Document Analysis
Source: Front Rehabil Sci. 2021 Oct 5;2:724589. doi: 10.3389/fresc.2021.724589 (PMC9397918; doi:10.3389/fresc.2021.724589)
Supplement: Supplementary file 1 [file Table_1.docx]

Supplementary Material

**Supplementary Table 1.** Description of resources that provide general information to siblings of individuals with a chronic health condition.

| **Booklets and books** |
| --- |
| Books were available for siblings and families, in which some books highlighted the importance of understanding the importance of sibling relationships. At one children’s hospital, young siblings can refer to a booklist such as a book told from the perspective of a main character who has questions when their sibling has a CHC and is in the hospital (1). One sibling wrote a book to chronicle her sibling relationship in growing up with her sister with a CHC. In addition, booklets are available to provide guidance to parents and teachers about how to communicate with siblings of someone with a CHC. For example, there was a booklet that described how to create inclusive classrooms and educate children about autism with a list of resources available specific for siblings (2). |
| **Podcast** |
| The stories of sibling relationships are also shared through other forms of media. There was a podcast from the United Kingdom (53) that was recommended, which tells the stories of individuals with a CHC, and one episode shared the stories of siblings in families with a child with a CHC. Personal stories from families, including siblings, are primarily shared through films and videos. Documentary films described the journey of the whole family, and while they only focused on the perspective of parents, there were stories about the relationship between the siblings and child with a CHC. |
| **Programs and workshops** |
| There are advertisements that announced past programs and workshops that were available to families. There were five workshops that were advertised as one-time events, in which one workshop was held for free in-person and organized at a children’s hospital in Ontario (105), and four documents from one children’s hospital that advertised workshops organized by another organization for siblings who are young carers ages 15-25 years old, with the option for virtual attendance and did not indicate a fee (106). Documents also advertised programs that were available for siblings if they preferred to participate in an extended activity for a few weeks, and advertised on an ongoing basis from four hospitals and two service providers in Ontario [references provided for the websites of ongoing advertisements for these programs, (54–59)]. Among the 34 documents of program advertisements, 16 were in-person, 6 were virtual due to COVID-19, and 12 did not indicate the type of program format. Furthermore, out of the 34 documents, there were 6 documents from two children’s hospitals (55,56) that referred to other available programs for siblings. There were 2 sibling support programs that were identified to be free, 2 programs that required a fee, and 9 programs that did not indicate a fee. Support involved not only workshops or programs, but also family events where the whole family was welcome to attend. A children’s treatment network hosted these family events at different geographical locations such as preschool playgroups, escape room activity, arts and crafts, and board games, with 6 events that required a nominal fee and 3 events that did not indicate a fee out of a total of 9 advertised events [reference to ongoing advertisements on the website (54)]. |
| **News articles** |
| ***Stories*** |
| Stories were published as news articles that were written either by parents (n=1), mothers (n=2) or a sibling (n=3), both a mother and sibling (n=1). Mothers focused on stories of their child’s lived experience, parenting multiple children with a CHC or the same CHC, and/or the roles that other children may assume when there is a child with a CHC. One mother described how her son had the role of being a playmate and supporter, in which her son played therapy games to ensure that her daughter practiced skills from a therapy intervention program (3). The primary topics of the articles authored by siblings included sharing their emotions about their sibling relationship, providing support with healthcare management, and transitioning into different roles as a sibling such as a caregiver. For some siblings, they shared their grief when a sibling with a CHC passed away (4). In some sibling relationships, both siblings had a CHC and they shared how they supported each other with managing each other’s healthcare. For example, two siblings had the same CHC and a sister shared her positive experience at a health clinic and encouraged her sister to attend the same clinic (5). Siblings shared their experiences with new roles that they had, such as being a full-time caregiver when the parents were no longer able to. One sister shared that she learned how to be a caregiver by experience, in which she had to find supports, organize finances, and identify funding from the government for her brother with a CHC (6). |
| ***Announcements about initiatives*** |
| News articles also published announcements about initiatives that were inspired by the stories of siblings. For example, an event was organized by an organization to provide an opportunity for young carers, siblings, along with professionals, policy makers and researchers to have discussions about priorities and actions to support young carers (107). A special event was also held to showcase the products from a program. Young people, both youth with cancer and their siblings participated in a Photovoice program to share their experiences of cancer, and they displayed their photographs during Childhood Cancer Awareness Month at a hospital (108). Six news articles (69–74) shared about different awareness days and months about specific disabilities and health conditions, and siblings were encouraged to share stories about their brother or sister with a CHC on these awareness days and months. |
| ***Research studies*** |
| The news articles also advertised research studies that were conducted about siblings [n=10, references provided for 8 articles (79,81–83,85,86,109,110), with 2 articles no longer posted on the website, but was included in the data analysis]. The topics of these research studies ranged from genetic studies that were conducted for specific health conditions, such as autism spectrum disorder and lymphoma, to successful organ transplants between siblings, to rehabilitation studies about the effectiveness of assistive equipment. There were also four news articles that advertised about current research studies that were actively recruiting sibling participants from one children’s hospital in Ontario (88) and one health center in Nova Scotia (87) at the time of publication, such as early intervention studies as well as a survey to understand the needs and feelings of siblings of youth with a CHC. |

References

1. Children’s Hospital - London Health Sciences Centre. Hematology and oncology [Internet]. 2021 [cited 2021 May 30]. Available from: https://www.lhsc.on.ca/hematology-oncology

2. Timmons V, Breitenbach M, MacIsaac M. Educating children about autism in an inclusive classroom [Internet]. 2016 [cited 2021 Jun 2]. Available from: https://www.princeedwardisland.ca/en/publication/educating-children-about-autism-inclusive-classroom

3. Provincial Health Services Authority. Rosalind’s story [Internet]. [cited 2021 May 30]. Available from: http://www.phsa.ca/health-info/hearing-loss-early-language/stories-from-families/rosalinds-story

4. Holland Bloorview Kids Rehabilitation Hospital. Isn’t a person more than a brain? [Internet]. 2013 [cited 2021 May 30]. Available from: http://bloom-parentingkidswithdisabilities.blogspot.com/2013/07/isnt-person-more-than-brain.html

5. Hamilton Health Sciences. Opting for an ostomy: a young woman’s journey with Crohn’s [Internet]. 2017 [cited 2021 Mar 12]. Available from: https://www.hamiltonhealthsciences.ca/share/abby-colling/

6. Kinross L. How difficult could caregiving be? This sister found out [Internet]. 2016 [cited 2021 May 30]. Available from: http://bloom-parentingkidswithdisabilities.blogspot.com/2016/02/how-difficult-could-caregiving-be-this.html
